# Comparison of Vacancy Sink Efficiency of Cu/V and Cu/Nb Interfaces by the Shared Cu Layer

**DOI:** 10.3390/ma12162628

**Published:** 2019-08-18

**Authors:** Huaqiang Chen, Jinlong Du, Yanxia Liang, Peipei Wang, Jinchi Huang, Jian Zhang, Yunbiao Zhao, Xingjun Wang, Xianfeng Zhang, Yuehui Wang, George A. Stanciu, Engang Fu

**Affiliations:** 1 State Key Laboratory of Nuclear Physics and Technology, School of Physics, Peking University, Beijing 100871, China; 2 Institute for Advanced Nuclear Energy, College of Energy, Xiamen University, Xiamen 361102, China; 3 State Key Laboratory of Advanced Optical Communication Systems and Networks, Peking University, Beijing 100871, China; 4 Zhongshan Institute, University of Electronic Science and Technology, Zhongshan 528400, China; 5 Center for Microcopy-Microanalysis and Information Processing, University Politehnica of Bucharest, 313 Splaiul Independentei, 060042 Bucharest, Romania

**Keywords:** radiation damage, sink efficiency, multilayer composite, interface, vacancy

## Abstract

This paper provides a new method to compare and then reveal the vacancy sink efficiencies quantitively between different hetero-interfaces with a shared Cu layer in one sample, in contrast to previous studies, which have compared the vacancy sink efficiencies of interfaces in different samples. Cu-Nb-Cu-V nanoscale metallic multilayer composites (NMMCs) containing Cu/V and Cu/Nb interfaces periodically were prepared as research samples and bombarded with helium ions to create vacancies which were filled by helium bubbles. A special Cu layer shared by adjoining Cu/V and Cu/Nb interfaces exists, in which the implanted helium concentration reaches its maximum and remains nearly constant with a well-designed incident energy. The results show that bubble-denuded zones (BDZ) close to interfaces exist, and that the width of the BDZ close to the Cu/V interface is less than that of Cu/Nb interface. This result is explained by one-dimensional diffusion theory, and the ratio of vacancy sink efficiency between Cu/V and Cu/Nb interfaces is calculated. Conclusively, Cu/Nb interfaces are more efficient than Cu/V interfaces in eliminating vacancies induced by radiation.

## 1. Introduction

Nanoscale metallic multilayer composites (NMMCs) have been widely studied due to their ultra-high strengths and enhanced radiation damage tolerance. During the last decade, a number of NMMCs were fabricated and researched, including Cu/Nb [1,2,3], Cu/V [4,5,6,7,8,9], Cu/Co [10], Cu/W [11], Ag/Ni [12] Al/Nb [13] etc. Hetero-interfaces, which separate adjacent layers with different structures or chemistry elements in these NMMCs, are found to act as sinks for point defects [14,15,16] during ion irradiation. In order to characterize the ability of a specific interface in absorbing point defects, the quantity called “sink efficiency” was firstly proposed by Sutton and Balluffi [17]. Experimental and computational studies suggest that sink efficiency can depend strongly on the interface crystallography and chemistry [18]. Cu/Nb and Cu/V NMMCs are usually chosen as the research targets to investigate how hetero-interfaces eliminate radiation-induced point defects efficiently. However, few experiments [19] provide a quantitative measurement of sink efficiency up to now, in which Shimin Mao et al. successfully compared the sink efficiencies of three kinds of interfaces, Cu/Nb, Cu/V, and Cu/Ni, based on the effects of different interfaces on radiation-enhanced diffusion. In this paper, a new method, based on observing the distribution of radiation-induced defect clusters, is proposed to conveniently and visually compare and then reveal the sink efficiencies of different interfaces in the same sample. 

Cu/Nb and Cu/V NMMCs have two things in common. The first is that the Kurdjumov-Sachs (K-S) orientation relationship [20] exists in both Cu/Nb and Cu/V interfaces, i.e., FCC (face centered cubic) (111) // BCC (body centered cubic) (110) // interface, and FCC <1 1 0> // BCC <1 1 1>. The other is the commonly used Cu element. Thus, we propose to take Cu as the intermediate layer connecting the Cu/V interface and Cu/Nb interface. If the two interfaces behave in the same way, the defects in Cu layer are expected to be distributed uniformly. However, interfaces play a big role in the distribution of defect clusters in their vicinity zones [20,21]. By means of observing the distribution of radiation-induced defect clusters in this intermediate Cu layer, we attempt to investigate the difference between Cu/V and Cu/Nb interfaces, and to determine the extent of the difference. 

## 2. Experiments

Cu-Nb-Cu-V NMMCs were prepared using the DC magnetron sputtering technique at room temperature on Si (100) substrates with individual layer thickness of 48 nm and total thickness of 576 nm (referred to as Cu-Nb-Cu-V 48 nm thereafter). In the deposition process, a base pressure of 4 × 10^−5^ Pa was reached prior to deposition and argon partial pressure during sputtering was kept at 0.5 Pa. The deposition rate was approximately 0.2 nm/s.

**Figure 1:**
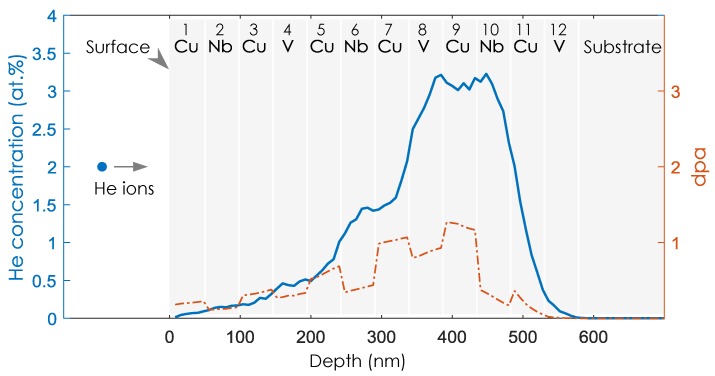
The depth distribution of helium concentration and dpa distribution in Cu-Nb-Cu-V 48 nm NMMCs simulated by SRIM. The helium ions are injected perpendicularly to the surface layer which is made of Cu and numbered as the 1st layer. The NMMCs are repeated of Cu-Nb-Cu-V periodically and the bottom layer is numbered as the 12th layer. The energy of the incident helium ions is 119 keV, and the total dose is 6 × 10^16^ ions/cm^2^.

The Stopping and Range of Ions in Matter (SRIM) [22] computer program developed by J. Ziegler and based on the Monte Carlo method was used to calculate the depth profile of He concentration in Cu-Nb-Cu-V NMMCs irradiated by He ions at an energy of 119 keV and a total dose of 6 × 10^16^ ions/cm^2^. Reasonably, the target model in SRIM simulation for Cu-Nb-Cu-V 48 nm NMMCs is the multilayered model, as Figure 1 shows, exactly corresponding to the layered morphology of which the multilayered composite consists. This model is used in the SRIM simulations of multilayered composites, differently from the compound model used in previous studies [1,2,3,4,5,6,7,8,9,10,11,12,13] in which multilayered composites are regarded as compounds. Compared to the compound model, the multilayered model highlights the presence of every individual layer with a corresponding chemical element. Therefore, the profile of the He concentration in every individual layer can be obtained. The threshold displacement energies of 30 eV for Cu, 40 eV for V and 60 eV for Nb [23] are chosen in the SRIM simulation.

Helium ions were chosen to radiate samples to create point defects inside with an incident energy of 119 keV. The injection dose of 6 × 10^16^ ions/cm^2^ was conducted at room temperature to make sure that the He concentration exceeded a critical value so that the defect clusters could be observed in TEM, as described in previous studies [6]. The temperature was almost constant during the He ion irradiation, with a negligible fluctuation of less than 2 °C. The cross-sectional transmission electron microscopy (XTEM) specimens were prepared in a sequential manner of grinding, Ar ion milling and low energy Ar ion polishing. Then, the microstructures of Cu-Nb-Cu-V NMMCs before and after irradiation were characterized using a FEI Tecnai F30 transmission electron microscope.

## 3. Results

The depth distributions of helium concentration (DHC) and displacements per atom (dpa) in Cu-Nb-Cu-V 48 nm NMMCs are predicted by SRIM, as Figure 1 shows. The He ions are injected perpendicularly to the surface layer, which is made of Cu and numbered as the 1st layer. The simulation predicts that the He concentration will initially increase with the increase of the penetration depth, before reaching a peak value of ~3.1 at.% at the beginning of the 9th Cu layer. Importantly, it remains nearly constant in the whole of the 9th Cu layer with a fluctuation of only ±0.1 at.%. This is due to the well-selected incident helium energy. Then, it decays over the Cu/Nb interface. Generally, the helium concentration is symmetrically distributed in the region composed of the 8th V layer, the 9th Cu layer and 10th Nb layer. The helium concentration reduces to zero at the interface between the bottom vanadium layer and silicon substrate, meaning that all of these implanted helium atoms stay in the nanolayered composite. 

**Figure 2:**
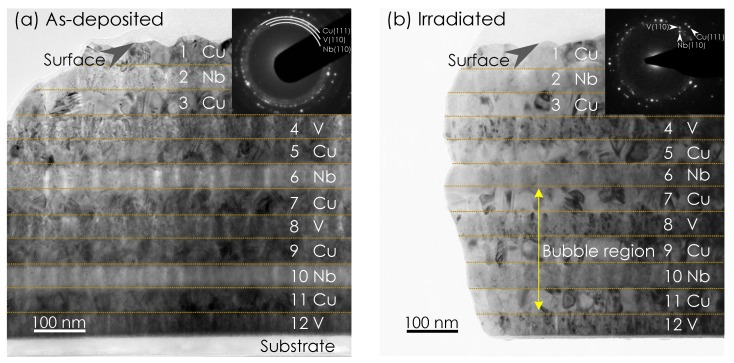
The bright-field XTEM images and corresponding SAED images of Cu-Nb-Cu-V 48 nm NMMCs in conditions of (**a**) as-deposited, (**b**) irradiated at room temperature with a dose of 6 × 10^16^ ions/cm^2^ at under-focused condition. In (**b**), bubbles are observed in the region of 7th to 11th layers and called as “Bubble region”.

The in-focused XTEM micrographs of as-deposited Cu-Nb-Cu-V 48 nm NMMC are presented in Figure 2a. The sample was composed of 12 layers in a periodical sequence of Cu-Nb-Cu-V from surface to substrate. In order to conveniently identify the layers in the nanolayered composite, each layer is numbered sequentially. The layer below the surface is numbered as the first layer, and that attached to the substrate is given as the 12th layer. The surface fluctuates because of some wear and tear during sample preparation, due to cutting and polishing. The thicknesses of these layers is almost uniform, at around 48 nm. The layered morphology and the selected area electron diffraction (SAED) image posted in the top-right corner collectively suggest that a strong texture structure with K-S orientation relationships exits, i.e., Cu (111)//V (110)//Nb (110), Cu <110>//V <111>//Nb <111>.

Figure 2b displays the under-focused TEM image of Cu-Nb-Cu-V 48 nm NMMCs irradiated at room temperature with a total dose of 6 × 10^16^ ions/cm^2^. Bubbles are observed in the region of 7th to 11th layers. These bubbles filled by helium atoms may be imaged by structure factor contrast under dynamical or bright-field kinematical imaging conditions [24]. The contrast mechanism is similar to that for disordered zones in ordered alloys or amorphous zones in crystalline matrices. Changing the focus from over focus to under focus, bubbles appear dark to bright, respectively. In this way, the existence of bubbles is confirmed. Setting the focus under a proper value, bubbles can be observed in an optimal contrast, i.e., they are too small to be seen at this magnification. Therefore, we didn’t indicate any bubbles individually in Figure 2b as typical examples. Alternatively, we have indicated the region where bubbles mainly exist in Figure 2b, and called it the “Bubble region”. The morphologies of both Cu/V and Cu/Nb interfaces in Cu-Nb-Cu-V 48 nm NMMCs remain immiscible after irradiation. The SAED images suggest that the K-S orientation relationships still exits after He ion irradiation, but it is slightly weaker than that of the as-deposited material.

**Figure 3:**
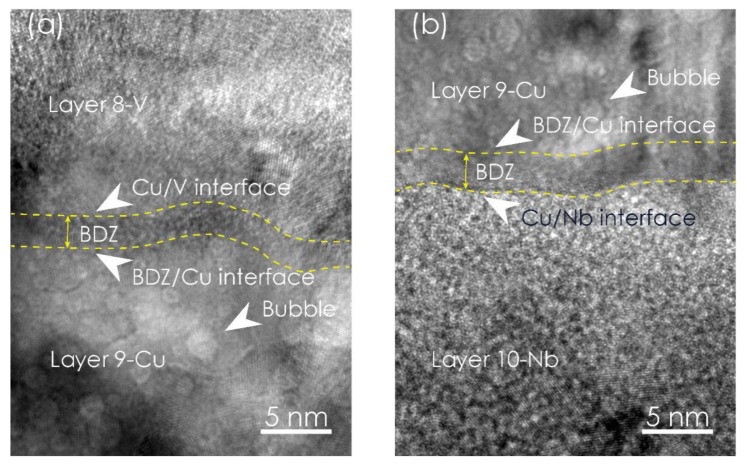
The typical HRTEM images of (**a**) the Cu/V interface sandwiched by the 8th V layer and the 9th Cu layer and (**b**) the Cu/Nb interface sandwiched by the 9th Cu layer and the 10th Nb layer in irradiated Cu-Nb-Cu-V 48 nm NMMC at room temperature with a dose of 6 × 10^16^ ions/cm^2^. The average width of the bubble denuded zone near the Cu/V interface in the 9th Cu layer is 2.5 ± 0.2 nm, while 3.5 ± 0.3 nm near the Cu/Nb interface in the 9th Cu layer. Bubbles are indicated by arrows.

Figure 3 shows the typical microstructures of Cu/V and Cu/Nb interfaces adjoining the 9th Cu layer in Cu-Nb-Cu-V 48 nm which was irradiated at room temperature with a dose of 6 × 10^16^ ions/cm^2^. The existence of the bubbles is confirmed and indicated by arrows. As we can see, bubbles are observed in Layer 9-Cu, but not in the 8-V and 9-Nb layers. Meanwhile, two zones free of bubbles in Layer 9-Cu close to the Cu/Nb and Cu/V interfaces exist. These zones, depleted of defect clusters near the interfaces, are referred to as ”bubble denuded zones” (BDZs) [20,21]. The BDZ boundaries were checked based on their definition and followed by the reported study [21]. By undertaking five measurements at different positions, the average width of the BDZ in the Layer 9-Cu close to Cu/Nb interface was determined to be 3.5 ± 0.3 nm, and 2.5 ± 0.2 nm for Cu/V interface. 

## 4. Discussion

In order to compare the sink efficiencies of Cu/V and Cu/Nb interfaces in the same sample for the first time, Cu-Nb-Cu-V NMMCs containing Cu/V and Cu/Nb interfaces are composed in a single sample for this paper. Previous studies have stated that the interfaces play a big role in the distribution of defect clusters in their vicinity zones [20,21]. Therefore, the Cu layer sandwiched by the V and Nb layers in Cu-Nb-Cu-V NMMC can be treated as an indication layer. The distribution of defect clusters in this Cu indication layer indicates the difference, and its extent difference, between the Cu/V and Cu/Nb interfaces. 

The most primary form of atomic damage sustained by an irradiated material is point defects, including vacancies and interstitials. These interstitials within the intermediate Cu layer after irradiation include helium atoms and disordered Cu atoms. As helium ion irradiation occurs over time, point defects in NMMCs gradually evolve into defect clusters. For Cu-Nb-Cu-V 48 nm NMMCs, bubbles are observed at room temperature, as Figure 2b shows. As a result of the constantly distributed helium concentration shown in Figure 1, the shared 9th Cu layer can behave as a stage by which display the strength of neighboring two Cu/V and Cu/Nb interfaces in eliminating point defects. Following the idea mentioned earlier, we focus on the bubbles in the 9th Cu layer. As Figure 3 shows, the bubbles are small and crowded at room temperature. The BDZ in the 9th Cu layer can be noticed and the average width of BDZ in the 9th Cu layer close to Cu/V interface is $\lambda_{\mathrm{Cu}/V}=2.5\pm 0.2\;\mathrm{nm}$, while $\lambda_{\mathrm{Cu}/\mathrm{Nb}}=3.5\pm 0.3\;\mathrm{nm}$ for Cu/Nb interface. The difference in BDZ width between these two interfaces may originate from the different interaction degrees of these two interfaces with radiation defects produced during helium bombardment; a detailed discussion is provided in the following paragraphs. 

In the formation stage of helium bubbles, vacancies are firstly formed and then combined with helium atoms into a stable cluster. These clusters may build up by capturing vacancies and helium atoms, and gradually become detectable large bubbles. Hence, the difference in width of these two BDZs originates from different vacancy sink efficiencies of interfaces. Following a previous study [16], the vacancy concentration in the vicinity of an interface with arbitrary vacancy sink efficiency η may be written as:

(1) $$c(x)=c_{eq}+\frac{K_{0}}{K_{s}}\left(1-\eta e^{-x\sqrt{\frac{K_{s}}{D}}}\right)$$

where x is the position along the direction normal to the interface plane, c is the vacancy concentration, *c_eq_* is the vacancy concentration under thermal equilibrium conditions and D is the vacancy diffusivity. $K_{0}$ is the rate of vacancy generation under radiation, which can be obtained using the Norgett-Ronbinson-Torrens model [25] with the help of SRIM. $K_{s}$ is the vacancy reduction rate in some kind of medium. In Figure 3a, the interface at x=0 is exactly referred to the Cu/V interface. Similarly, in Figure 3b, the interface at x=0 is exactly referred to the Cu/Nb interface. As is well known, a critical vacancy concentration is necessary when supersaturated vacancies in matrix precipitate into bubbles. Thus, the vacancy concentration in the BDZ/Cu interface near Cu/V is the critical vacancy concentration to form bubbles; we denote its value as $c_{b}$. From Equation (1), we can see that the distance corresponding to the critical vacancy concentration is exactly the width of the BDZ, i.e., $c\left(x=\lambda_{\mathrm{Cu}/V}\right)=c_{b}$. Substituting $c_{b}$ and $\lambda_{\mathrm{Cu}/V}$ into Equation (1), and rewriting the equation, we get the vacancy sink efficiency for the Cu/V interface:

(2) $$\eta_{Cu/V}=\frac{1-\left(c_{b}-c_{eq}\right)K_{s}/K_{0}}{e^{-\lambda_{Cu/V}\sqrt{\frac{K_{s}}{D}}}}$$

Because the BDZ close to Cu/Nb interface and the BDZ close to Cu/V interface exist in the same Cu layer, the vacancy concentration in the BDZ/Cu interface near Cu/Nb interface takes the same value of $c_{b}$. So, for Cu/Nb interface, the sink efficiency is:

(3) $$\eta_{Cu/\mathrm{Nb}}=\frac{1-\left(c_{b}-c_{eq}\right)K_{s}/K_{0}}{e^{-\lambda_{Cu/Nb}\sqrt{\frac{K_{s}}{D}}}}$$

Note that the numerators of these two sink efficiencies are exactly same. So, the ratio of sink efficiencies of these two interfaces is:

(4) $$\alpha =\frac{\eta_{Cu/V}}{\eta_{Cu/Nb}}=e^{\sqrt{\frac{K_{s}}{D}}\left(\lambda_{Cu/V}-\lambda_{Cu/Nb}\right)}$$

The rate of recombination $K_{s}$ takes the value of $5\times 10^{6}\;s^{-1}$ [26]. The vacancy diffusivity $D_{v}$ is computed as $D\;=1.938\times 10^{-9}\;m^{2}\;s^{-1}$ [14]. Taking all the values of these listed parameters in the equation above, we get α=0.95, i.e., the vacancy sink efficiency of Cu/V interface is only 95% of Cu/Nb. 

Conclusively, the Cu/Nb interface had a higher sink efficiency than that of the Cu/V interface. The difference in vacancy sink efficiency for Cu/V and Cu/Nb interfaces may be attributed to defect trapping sites in them. Misfit dislocation interactions (MDIs) have been shown to serve as preferential vacancy trapping sites at some hetero-interfaces in previous studies. And the Cu/Nb interface contains a 5 times greater density of MDIs than Cu/V [16]. Therefore, The Cu/Nb interface prevails over the Cu/V interface in eliminating vacancies, suppressing the formation of helium-vacancy clusters and making bubble-denuded zones slightly wider in their vicinities. 

## 5. Conclusions

In this paper, a new method to quantitively compare the vacancy sink efficiencies between different interfaces is proposed. This method is the first to integrate the comparison in the same sample, in contrast to the previous studies which have compared two interfaces existing in two individual samples. The average width of bubble-denuded zones was measured to be 2.5 ± 0.2 nm for the Cu/V interface and 3.5 ± 0.3 nm for the Cu/Nb in our designed experiment. Based on one-dimensional diffusion theory, the ratio of vacancy sink efficiencies of Cu/V and Cu/Nb interfaces was shown to be approximately 95% of Cu/Nb, indicating that Cu/Nb interfaces are slightly more efficient than Cu/V interfaces in eliminating point defects and associated He bubbles induced by radiation.

## Data Availability

All data included in this study are available upon request by contact with the corresponding author.
